# Supplementary material for: Epidemiological and clinical characteristics of scrub typhus in Guizhou Province, China: An outbreak study of scrub typhus
Source: PLoS Negl Trop Dis. 2024 Mar 5;18(3):e0011963. doi: 10.1371/journal.pntd.0011963 (PMC10914282; doi:10.1371/journal.pntd.0011963)
Supplement: S1 Table — (PDF) [file pntd.0011963.s004.pdf]

S1 Table

GenBank numbers of *Orientia tsutsugamushi* sequences obtained in this study.

Isolate	56-kDa	47-kDa	16S rRNA
Orientia tsutsugamushi strain Guizhou1	OR513496	OR513503	OR501559
Orientia tsutsugamushi strain Guizhou3	OR513497	OR513504	OR501560
Orientia tsutsugamushi strain Guizhou5	OR513499	OR513505	OR501561
Orientia tsutsugamushi strain Guizhou6	OR513500		OR501562
Orientia tsutsugamushi strain Guizhou7	OR513501	OR513506	OR501563
Orientia tsutsugamushi strain Guizhou8	OR513498	OR513507	OR501564
Orientia tsutsugamushi strain Guizhou-mouse	OR513502	OR513508	OR501565
