# Supplementary material for: Epidemiological and clinical characteristics of scrub typhus in Guizhou Province, China: An outbreak study of scrub typhus
Source: PLoS Negl Trop Dis. 2024 Mar 5;18(3):e0011963. doi: 10.1371/journal.pntd.0011963 (PMC10914282; doi:10.1371/journal.pntd.0011963)
Supplement: S2 Table — (PDF) [file pntd.0011963.s005.pdf]

S2 Table

Primers used for the Scrub typhus.

Name	Gene	Sequence5’-3’	Length(bp)	Reference
Ric-F	16S	5-YTACGGAATAACTTTTAGAAA-3	900	[1]
Ric-R1	16S	5-CATGATGACTTGACRTCGT-3		
Ric-R2	16S	5-CATCTCACGACACGAGCTG-3		
56kDex5	56kDa	5-WAAACTTACACCWCCTCARCCT-3	600	
56kDin5	56kDa	5-CCTATAAGYAYWGCKGATCGTG-3		
56kD3	56kDa	5-GCWGCYGCTRCTGCWTCTTG-3		
47kDex5	47 kDa	5-CATTACAAGGYATAAGTAATGT-3	800	
47 kDin5	47 kDa	5-GGAACAGTAACWAATGGYATT-3		
47 kDin3	47 kDa	5-AAGCAATGTAAAGCATTCTATCT-3		

Reference

[1] Lu M, Zhou D, Xie S, Wang Y, Jiang L, Wang W, et al. Genetic recombination of *Orientia tsutsugamushi* strains from scrub typhus patients in Guangxi, Southwest China, and the analysis of clinical features. Microbes Infect. 2023;25(5):105098. doi: 10.1016/j.micinf.2023.105098. PMID: 36621527.
