# Supplementary material for: Epidemiological and clinical characteristics of scrub typhus in Guizhou Province, China: An outbreak study of scrub typhus
Source: PLoS Negl Trop Dis. 2024 Mar 5;18(3):e0011963. doi: 10.1371/journal.pntd.0011963 (PMC10914282; doi:10.1371/journal.pntd.0011963)
Supplement: S1 Text — (PDF) [file pntd.0011963.s006.pdf]

S1 Text

Case definition

1 Epidemiological history: a person who has been in or visited a tsutsugamushi endemic area within 3 weeks prior to the onset of the disease and has a history of field activities.

2 Clinical manifestations:

2.1 A person had clinical symptoms of fever;

2.2 A person had lymphadenopathy;

2.3 A person had skin rash;

2.4 A person had specific eschar or ulcers.

3 Laboratory tests:

3.1 single serum OX-K agglutination titer ≥ 1:160 in the WeilFelix test;

3.2 A 4-fold or greater increase in serum IgG antibodies during the acute and recovery phases, as measured by indirect immunofluorescence antibody detection (IFA).

3.3 Positive results of O. tsutsugamushi by PCR;

3.4 Isolation of tsutsugamushi pathogens from clinical samples.

The diagnostic criteria for suspected cases meet 1, 2.1 plus 2.2 or 2.3.

The diagnostic criteria for clinically confirmed cases meet suspected cases plus 2.4; or both 1, 2.1, and 2.4.

The diagnostic criteria for laboratory confirmed cases meet suspected cases plus 3.2 or 3.3 or 3.4; or meet clinically confirmed cases plus any one of 3.
