# Supplementary material for: Epidemiological and clinical characteristics of scrub typhus in Guizhou Province, China: An outbreak study of scrub typhus
Source: PLoS Negl Trop Dis. 2024 Mar 5;18(3):e0011963. doi: 10.1371/journal.pntd.0011963 (PMC10914282; doi:10.1371/journal.pntd.0011963)
Supplement: Attachment — Submitted filename: Response to reviewers (PNTD-D-23-01304).docx [file pntd.0011963.s008.docx]

Dear Editors and Reviewers:

Thank you for your letter and for the reviewers’ comments concerning our manuscript entitled “Epidemiological and clinical characteristics of scrub typhus in Guizhou Province, China: An outbreak study of scrub typhus” (PNTD-D-23-01304). Those comments are all valuable and very helpful for revising and improving our paper, as well as the important guiding significance to our researches.

According to the editor and reviewers’ comments, we have made extensive modifications to our manuscript and supplemented extra data to make our results convincing. In this revised version, changes to our manuscript were all highlighted within the document by using red colored text. Regarding the reviewer and editor's suggestion to shorten the article to include only the relevant aspects of this new focus of scrub typhus in this area, we have deleted and adjusted the materials and methods, discussion parts, and hope to get your approval.

**Responds to the reviewer’s comments:**

**Reviewer #1: PNTD-D-23-01304**

1. Correct all typo errors after a thorough read through again by all authors.

Response: We feel sorry for our carelessness. In our resubmitted manuscript, all the authors have read through the article and made corrections.

2. Use of words and digits to be made uniform throughout (like 1 or one).

**Response:** We sincerely thank the reviewer for their valuable feedback, and we have unified into digits. For example, Page 5, Lines 130: The 7 whole blood samples and 1 BALF sample were collected from 21 patients with fever and a history of outdoor activity.

3. Improve the overall presentation.

**Response:** We thank the reviewer for this valuable suggestion, and the whole manuscript has been polished accordingly. These changes will not influence the content and framework of the paper. And here we did not list the changes but marked in red in the revised paper. We appreciate for your warm work earnestly and hope that the correction will meet with approval.

**Reviewer #2: PNTD-D-23-01304**

1. Page 3, Lines 84 & 90: From July to August 2022, 21 cases of fever in residents of the Guizhou Province. In the same period, 84 mouse samples were collected.

Was any of the mice infected with Chigger mites? Was there any attempt to collect Chigger mites and any molecular analysis in those vectors?

**Response:** We gratefully appreciate for your comment. We attempted to collect Chigger mites during the sampling process, but unfortunately, we were unable to conduct molecular detection of chiggers as we did not collect them. In the following monitoring work, we will continue to collect the host and vector of *tsutsugamushi* disease in this area.

2. Page 4, Line 103-106: Case definition: The classification for suspected, clinically confirmed and laboratory-confirmed cases was based on the technical guidelines for the prevention and control of scrub typhus issued by the Chinese CDC [8].

The reference is not available to access. A summary of the case definition may be provided with a focus on all three groups. The same may be provided as a supplementary file.

**Response:** We feel sorry for the inconvenience brought to the reviewer. In response to this issue, we have provided detailed case definitions for suspected, clinically confirmed and laboratory confirmed cases in the form of supplementary information (Text S1).

Supplementary materials are as follows:

1 Epidemiological history: a person who has been in or visited a tsutsugamushi endemic area within 3 weeks prior to the onset of the disease and has a history of field activities. 2 Clinical manifestations:

2.1 A person had clinical symptoms of fever;

2.2 A person had lymphadenopathy;

2.3 A person had skin rash;

2.4 A person had specific eschar or ulcers.

3 Laboratory tests:

3.1 single serum OX-K agglutination titer ≥ 1:160 in the WeilFelix test;

3.2 A 4-fold or greater increase in serum IgG antibodies during the acute and recovery phases, as measured by indirect immunofluorescence antibody detection (IFA).

3.3 Positive results of O. tsutsugamushi by PCR;

3.4 Isolation of tsutsugamushi pathogens from clinical samples.

The diagnostic criteria for suspected cases meet 1, 2.1 plus 2.2 or 2.3.

The diagnostic criteria for clinically confirmed cases meet suspected cases plus 2.4; or both 1, 2.1,2.4.

The diagnostic criteria for laboratory confirmed cases meet suspected cases plus 3.2 or 3.3 or 3.4; or meet clinically confirmed cases plus any one of 3.

3. How these cases are defined as outbreaks? The cases and mouse samples were collected in a short span of 1-2 months. A pictorial representation of a line listing of the cases (with suspected cases, clinically confirmed cases and lab-confirmed cases) along with mouse samples during the period may be made and incorporated.

**Response:** We feel great thanks for your professional review work on our article. An outbreak is the sudden occurrence of many patients with the same symptoms over a short period of time in a local area or collective unit. The patients in this study were all in this area of Rongjiang County, and since there were no local cases of scrub typhus reported from the beginning of the direct network reporting in 2003, as well as the fact that the earliest and latest onset of illness in these patients was within two months, and that most of them presented with fever, chills, headache, and eschar or ulcer, these cases met the definition of an outbreak.

We agree with your suggestion, for the sampling time of cases and mice, we made the following figure (Page 4, Line 98-99):

**Fig 2. The number of cases and mouse collected in each period.**

**A red dot in the figure represents the collection period of a positive mouse.**

4. Need better quality images

**Response:** We have updated the sharpness of all the images in the article and processed them using the PACE tool.

**Data Availability:** The authors confirm that all data underlying the findings are fully available without restriction. All relevant data are within the paper and its Supporting Information files.

**References:** We have checked reference list and it is complete and correct.

Once again, we thank you for the time you put in reviewing our paper and look forward to meeting your expectations. Since your inputs have been precious, we would like to acknowledge your contribution explicitly. Look forward to hearing from you.

Yours sincerely,

Jia He (hejia@icdc.cn)

Correspondence to:

Prof. Tian Qin (qintian@icdc.cn)

National Key Laboratory of Intelligent Tracking and Forecasting for Infectious Diseases, Chinese Center for Disease Control and Prevention
