# Supplementary material for: Epidemiological and clinical characteristics of scrub typhus in Guizhou Province, China: An outbreak study of scrub typhus
Source: PLoS Negl Trop Dis. 2024 Mar 5;18(3):e0011963. doi: 10.1371/journal.pntd.0011963 (PMC10914282; doi:10.1371/journal.pntd.0011963)
Supplement: Attachment — Submitted filename: PNTD-D-23-01304 Reviewer Comment.docx [file pntd.0011963.s007.docx]

**Reviewer comment: PNTD-D-23-01304**

1. Page 3, Lines 84 & 90: From July to August 2022, 21 cases of fever in residents of the Guizhou Province. In the same period, 84 mouse samples were collected.

Was any of the mice infected with Chigger mites? Was there any attempt to collect Chigger mites and any molecular analysis in those vectors?

2. Page 4, Line 103-106: Case definition: The classification for suspected, clinically confirmed and laboratory-confirmed cases was based on the technical guidelines for the prevention and control of scrub typhus issued by the Chinese CDC [8].

The reference is not available to access. A summary of the case definition may be provided with a focus on all three groups. The same may be provided as a supplementary file.

3. How these cases are defined as outbreaks? The cases and mouse samples were collected in a short span of 1-2 months. A pictorial representation of a line listing of the cases (with suspected cases, clinically confirmed cases and lab-confirmed cases) along with mouse samples during the period may be made and incorporated.
